# Ultra-Performance Liquid Chromatography/Mass Spectrometry-Based Metabolomics for Discovering Potential Biomarkers and Metabolic Pathways of Colorectal Cancer in Mouse Model (ApcMin/+) and Revealing the Effect of *Honokiol*

**DOI:** 10.3389/fonc.2021.671014

**Published:** 2021-09-13

**Authors:** Xin Chen, Bo-lun Shi, Run-zhi Qi, Xing Chang, Hong-gang Zheng

**Affiliations:** ^1^Guang’anmen Hospital, China Academy of Chinese Medical Sciences, Beijing, China; ^2^China Academy of Chinese Medical Sciences, Beijing, China; ^3^Department of Oncology, Guang’anmen Hospital, China Academy of Chinese Medical Sciences, Beijing, China; ^4^Beijing University of Chinese Medicine, Beijing, China

**Keywords:** metabolomics, biomarkers, metabolites, ultra-performance liquid chromatography, mass spectrometry

## Abstract

Endogenous metabolites are a class of molecules playing diverse and significant roles in many metabolic pathways for disease. Honokiol (HNK), an active poly-phenolic compound, has shown potent anticancer activities. However, the detailed crucial mechanism regulated by HNK in colorectal cancer remains unclear. In the present study, we investigated the therapeutic effects and the underlying molecular mechanisms of HNK on colorectal cancer in a mouse model (ApcMin/+) by analyzing the urine metabolic profile based on metabolomics, which is a powerful tool for characterizing metabolic disturbances. We found that potential urine biomarkers were involved in the metabolism of compounds such as purines, tyrosines, tryptophans, etc. Moreover, we showed that a total of 27 metabolites were the most contribution biomarkers for intestinal tumors, and we found that the citrate cycle (TCA cycle) was regulated by HNK. In addition, it was suggested that the efficacy of HNK was achieved by affecting the multi-pathway system *via* influencing relevant metabolic pathways and regulating metabolic function. Our work also showed that high-throughput metabolomics can characterize the regulation of metabolic disorders as a therapeutic strategy to prevent colorectal cancer.

## Introduction

Intestinal tumors include benign tumors (e.g., adenomas) and malignant tumors such as colorectal cancer (CRC), which is a high-grade and fatal malignant disease that often results from adenomas (1), as well as inflammatory bowel disease (IBD) and Crohn’s disease (2–4). The inflammatory pathway is active in intestinal epithelial cells to promote the progression of cancer (5–7). Therefore, a handy, precise, and reliable means of diagnosis is particularly important for preventing tumorigenesis. Although gene prediction and routine screening methods such as colonoscopy, blood markers, and fecal occult blood tests have been served in the clinical practice, these methods are costly and invasive. Therefore, it is critical to establish a specific and noninvasive high-throughput method to solve this predicament.

Increasing evidence has shown that metabolism has a key role in health and disease (8). Metabolomics, one of the omics studies focused on small molecules, has a special high-throughput feature of providing an opportunity to perform a systematic investigation of the abnormal process of disease by presenting subtle biomarkers and pathways (9–12). Compared to the traditional detection method, the analysis samples were obtained more conveniently and non-invasively (13). Notably, urine is the biofluid of choice for the discovery of non-invasive biomarkers in metabolomic detection. Compared with other biological samples, urine is not difficult to collect and is rich in metabolites, which reflects physiological and pathological conditions *in vivo* (14–16). Mass spectrometry (MS) provides the best sensitivity, selectivity, and identification ability for analyzing the composition of most metabolites in urine samples (17). The hyphenated technique of MS has already been used to analyze biofluids in organisms.

In recent years, the global cancer burden, including the number of deaths, has increased due to the widespread cancer disease. Honokiol (HNK) is a natural biphenolic compound that has been used for its beneficial effects against tumor growth. Recent reports have shown that it can activate the pro-apoptotic factors, suppress anti-apoptotic protein and transcription factors, regulate various enzymes, and cell cycle proteins, and inhibit protein tyrosine kinases activity (18–20). However, the detailed crucial mechanism regulated by HNK in CRC is still unclear. The study aims to investigate the inhibitory effect of HNK on the development of intestinal tumors. Urine metabolomics techniques and multivariate statistical analysis are relatively comprehensive and non-stimulating ways to explain the changes in metabolites and metabolic pathways by treatment, and this verifies the effectiveness of HNK and explores potential targets of efficacy.

## Methods

### Experimental Animals

Male wild-type mice and APC^min/+^ mice were purchased from Nanjing Biomedical Research Institute of Nanjing University (Nanjing, China), all of which were 7 weeks old and fed for 2 weeks with water *ad libitum*. Based on weight, APC^min/+^ mice were randomly divided into the model group (n=10) and HNK-treated group (n=10). Since the 9th week, HNK (high-performance liquid chromatography, 99%; formula: C_18_H_18_O_2_) dissolved in dimethyl sulfoxide (DMSO) was orally administered to mice at the concentration of 400 mg/kg body weight for 10 weeks. The mice in the control group were administered DMSO orally. The experimental procedures were approved by the Animal Care and Ethics Committee, and all experiments were performed in accordance with the Declaration of Helsinki.

### Urine Collection and Preparation

Urine samples were gathered from 5 p.m. to 8 a.m. the next day for each mouse and collected every 2 days. The urine samples were placed in the 2.0 ml Eppendorf tubes, which were then centrifuged for 10 min (13,000 rpm, 4°C), and the impurities were removed. The supernatant was transferred to a 1.5 ml tube and stored at -80°C. Before analysis, the urine samples were thawed in an icy water bath at room temperature. Next, each urine sample was centrifuged for 10 min at 13,000 rpm (4°C). We transferred 100 μl urine into another tube and added 600 μl double distilled water, the self-made mixture was blended for 30s, and 0.45 µm was filtered. Finally, the filtrate was transferred into a sample cup.

### Chromatography and Mass Spectrometry Conditions

A chromatographic experiment was performed on an ACQUITY UPLCTM C18 column (Waters, USA, 1.7μm, 100mm×2.1 μm i.d.) in the ultra-high-performance liquid chromatography (UPLC) system (Waters Corp., Milford, MA/USA). The column temperature was maintained at 40°C. The flow rate was set at 0.4ml/min. The sample injection volume was 4µL. Solvent A was acetonitrile mixed with 0.1% formic acid, and solvent B was water mixed with 0.1% formic acid. The gradient elution programs were: 0min, ~99% B, 0-1min ~87% B, 1-2.5min ~70% B, 2.5-5min ~1% B, 5~7min ~99% B.

Electrospray ionization source (ESI) was used in the Synapt TM G2-Si mass spectrometry system (high-resolution quadrupole mass spectrometer). The flow and temperature of desolvation gas were 600 L/h and 350°C, respectively. The flow rate of the cone back flush gas was 50 L/h. Ion source temperature was 110°C and the voltage was set at 30 V. The capillary voltage was 3 kV. Online water quality correction was performed using the Locksray calibration system of Waters Corporation, USA. The calibrated substance was leucine-enkephalin ([M+H]^+^=556.2771, [M-H]^-^=554.2771). The mass scan range was 50~1000 Da, and the scan time was 0.2 s.

### Data Processing

Peak recognition, peak matching, peak alignment, and normalization were performed on the raw urine UPLC-MS data by using Progenesis QI 2.0 (Waters, USA). The data was imported into the MassLynx 4.1 for unsupervised component analysis (principal component analysis PCA) and supervised component analysis (partial least-squared discriminant analysis PLS-DA and partial least-squared discriminant analysis OPLS-DA), which supplied the intuitive diversity of the different groups. The following was used to identify significant biomarkers contributing to the group. Eligible ions needed to be satisfied with both VIP>1 from the OPLS-DA and p<0.05 from the t•test). The MS/MS fragment matched the databases: METLIN, HMDB, ChemSpider, and MASSBANK. The retention time was another corroborative reference for identification.

### Pathway Analysis and Network Prediction

MetaboAnalyst 3.0 (http://www.metaboanalyst.ca/) is a comprehensive tool suite for metabolomics data analysis. The module of ‘metabolic pathway analysis’ was applied in the present study to explore the associated metabolic pathways for all the differentiated metabolites. Additionally, the enrichment analysis can produce predicted metabolite sets and key regulatory information for metabolic mechanisms in the metabolomics data.

## Results

### Metabolic Profiling of the Urine Samples

According to the condition of the grouped chromatography, the urine samples were analyzed for the metabolic phenotype by the UPLC-Q-TOF-MS spectroscopy, and all the chromatograms of urine samples were obtained in the ESI+ (**Figure S1**) and ESI- (**Figure S2**). The spectra were stable, and the peaks were evenly distributed. The original “.raw” data were imported into Waters Progenesis QI 2.0 software for peak extraction, peak alignment, and picking peaks. Based on the HMDB, the 5719 ions under the positive ion mode and 6108 ions under the negative ion mode were obtained. There were some differences among different metabolomic profiling, and we used multiple pattern recognition methods to deal with the data.

### The Identification of Potential Biomarkers of APC^min/+^ Transgenic Mice Model

**Figure 1:**
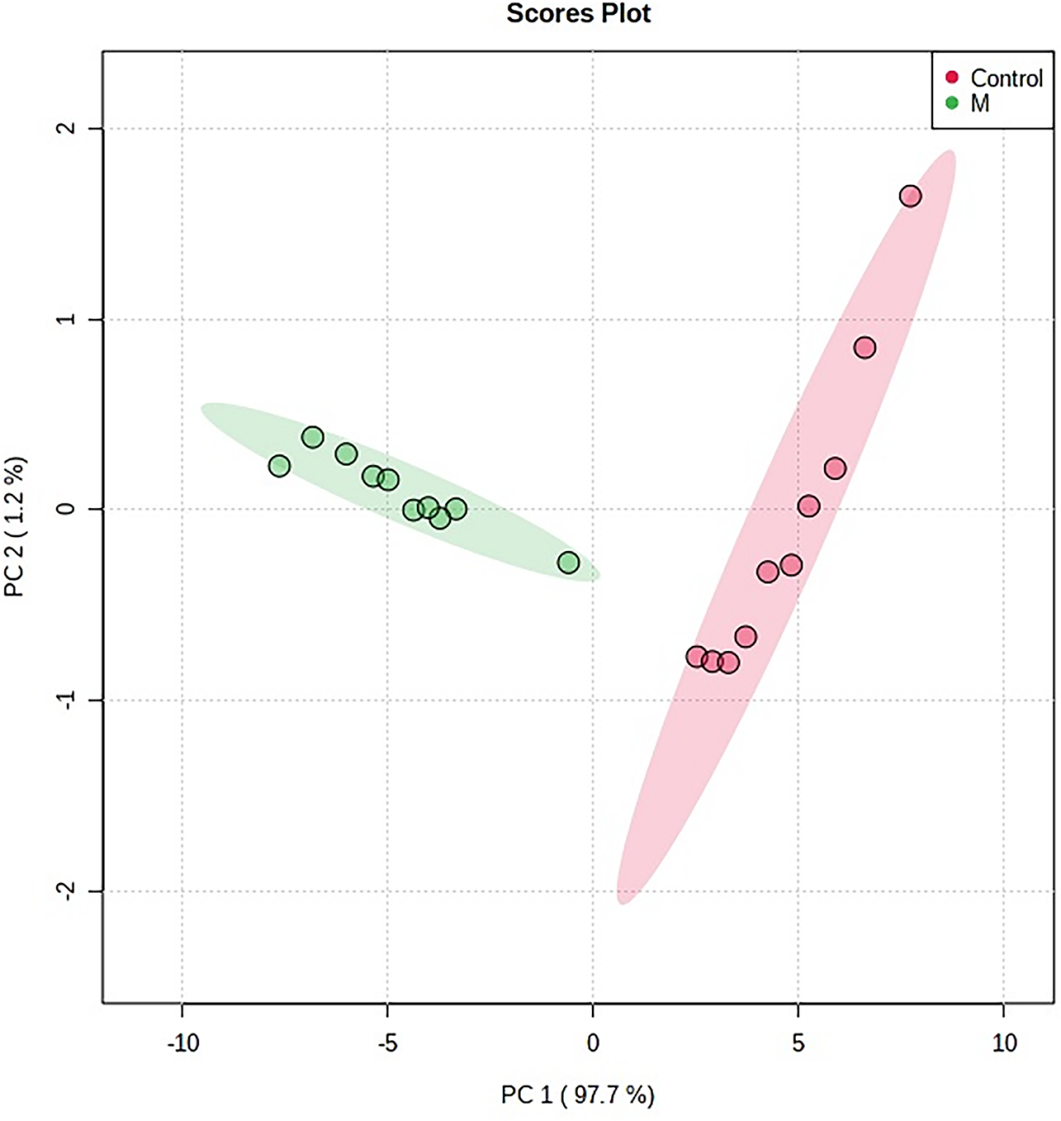
The PCA scores plot of urine samples from the control group and the model group. M, model group. The red dot indicates the control group, and the green dot indicates the model group.

**Figure 2:**
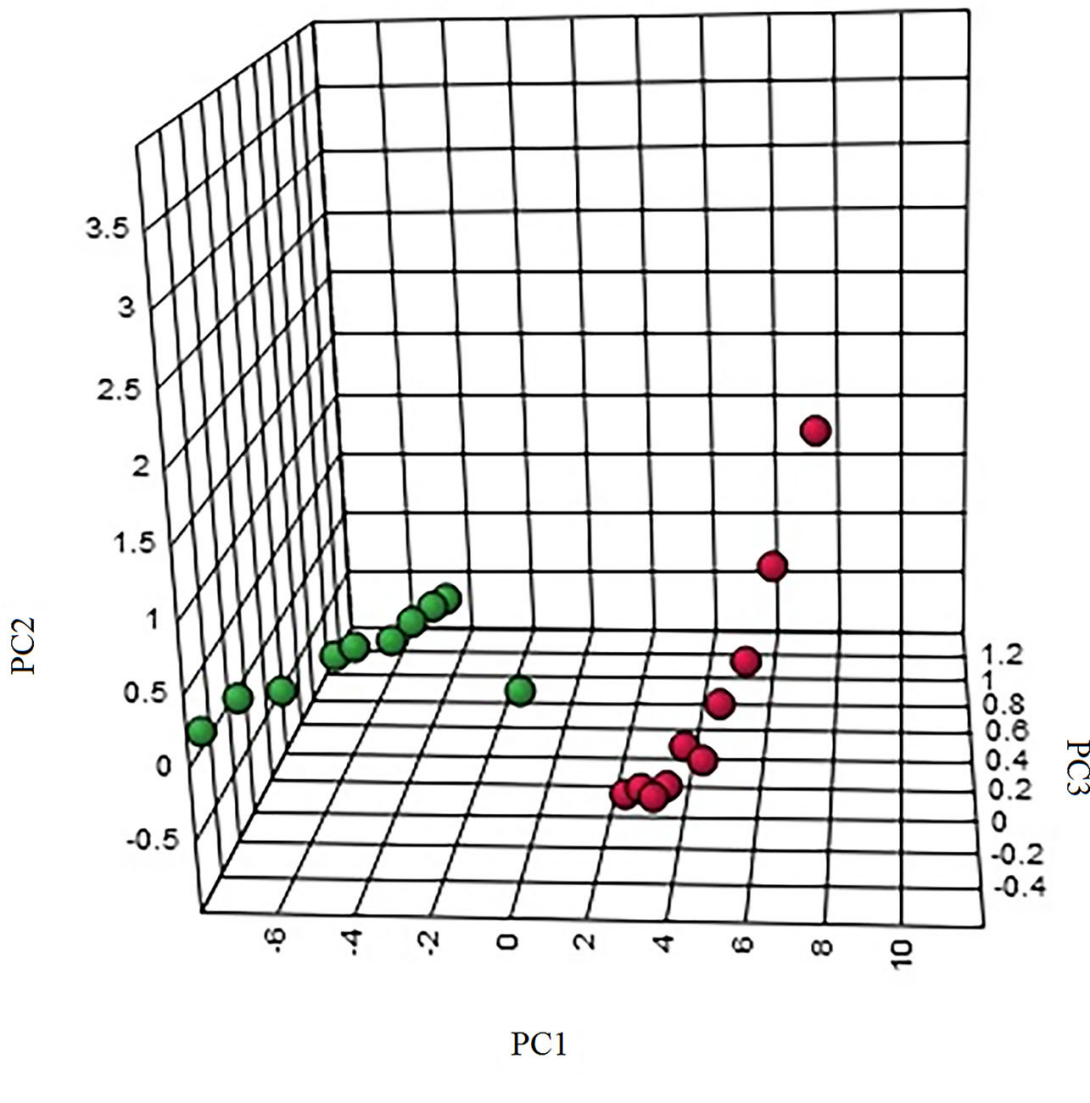
The three-dimensional (3D) score plot of the urine samples from the control group and the model group. M, model group. The red dot indicates the control group, and the green dot indicates the model group.

**Figure 3:**
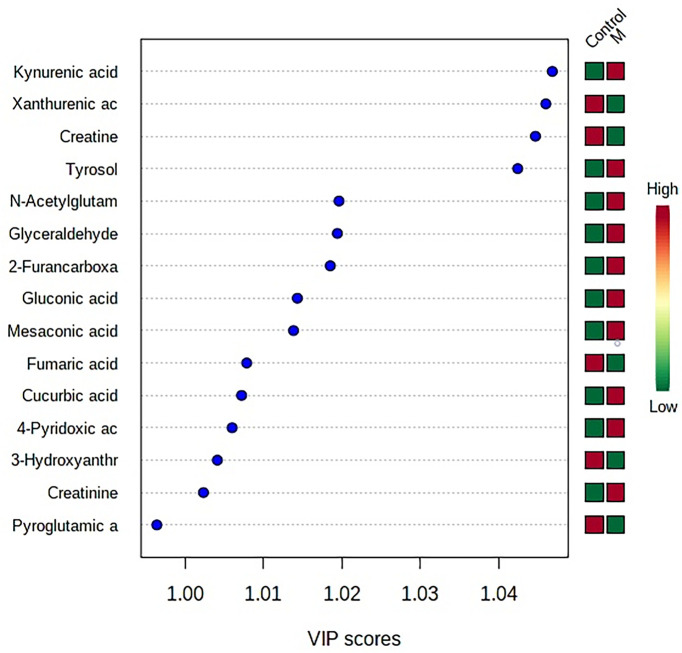
VIP coefficient of variation of OPLS-DA for screening conditions for the potential biomarkers. “Low/high” refer to high expression and low expression. M, model group.

**Figure 4:**
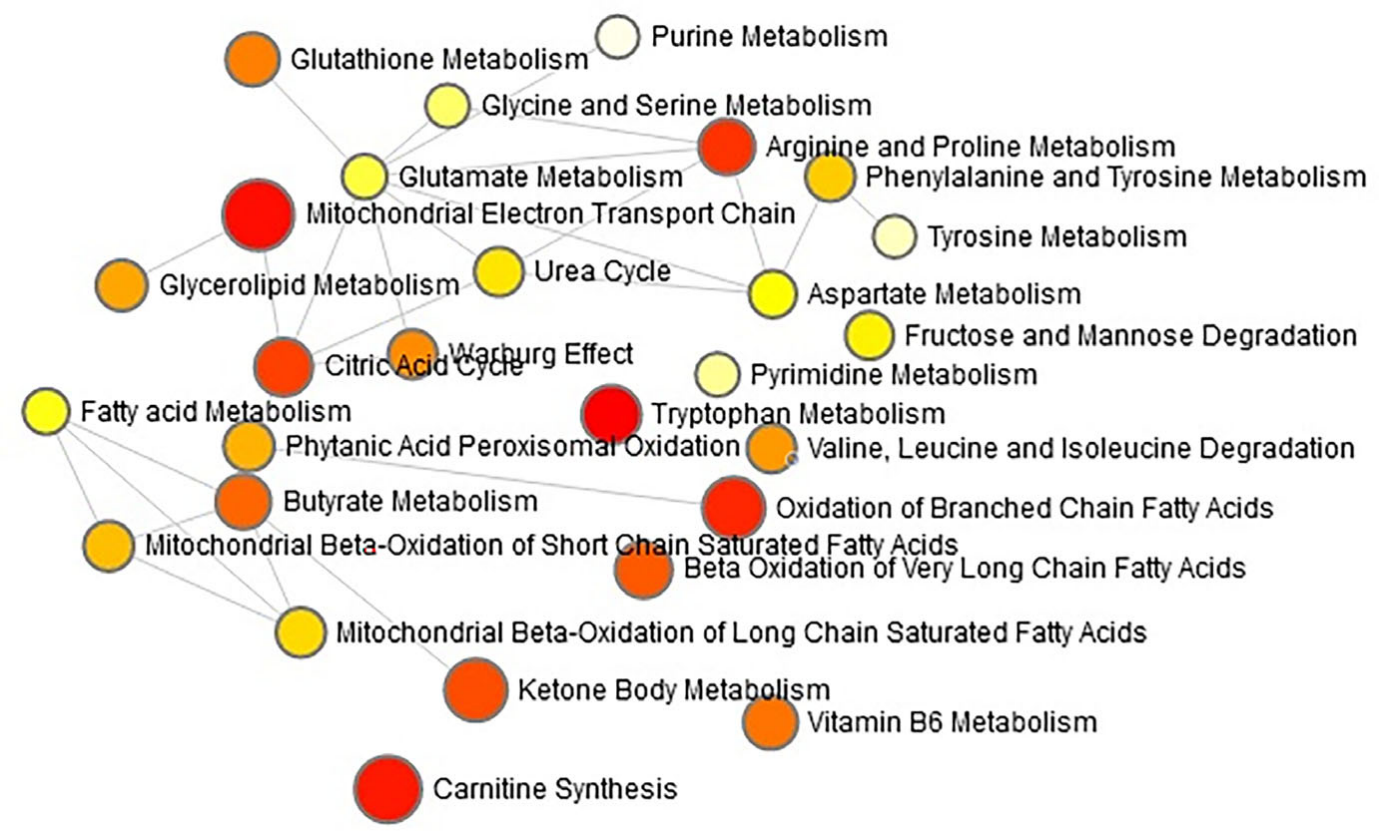
The network pathways analysis in the APCmin/+ mice by the MetaboAnalyst 4.0 tool. The module of ‘Metabolic pathway analysis’ was applied in the present study to explore the associated metabolic pathways for all the differentiated metabolites. Each circle indicates a significantly altered cluster of metabolites.

**Figure 5:**
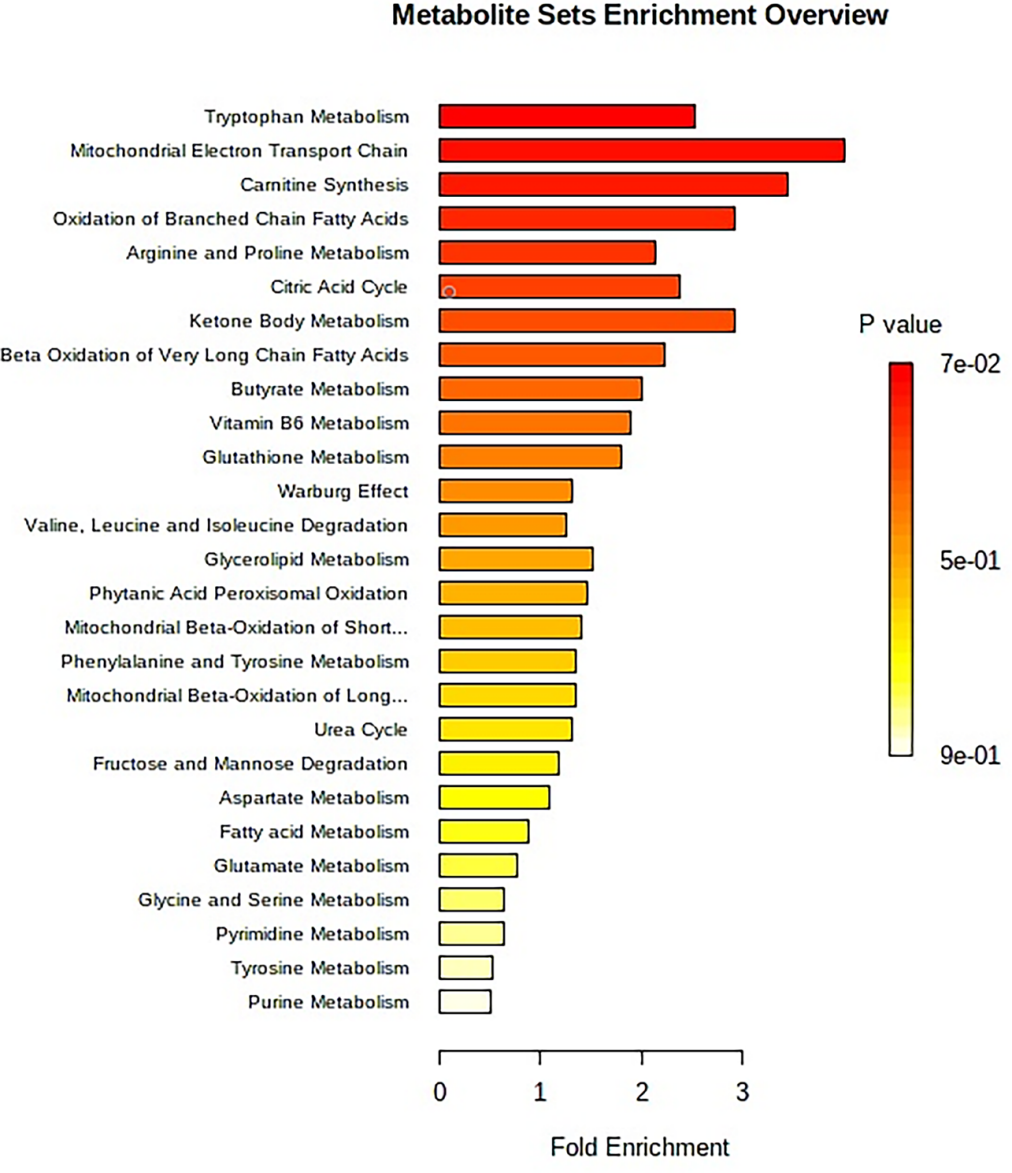
The metabolite sets enrichment overview of urine samples for APCmin/+ mice. The enrichment analysis produces the predicted metabolite sets and key regulatory information to metabolic mechanism in this study. An overview of metabolite sets by the enrichment analysis is reported in **Supplementary Table S2**.

**Figure 6:**
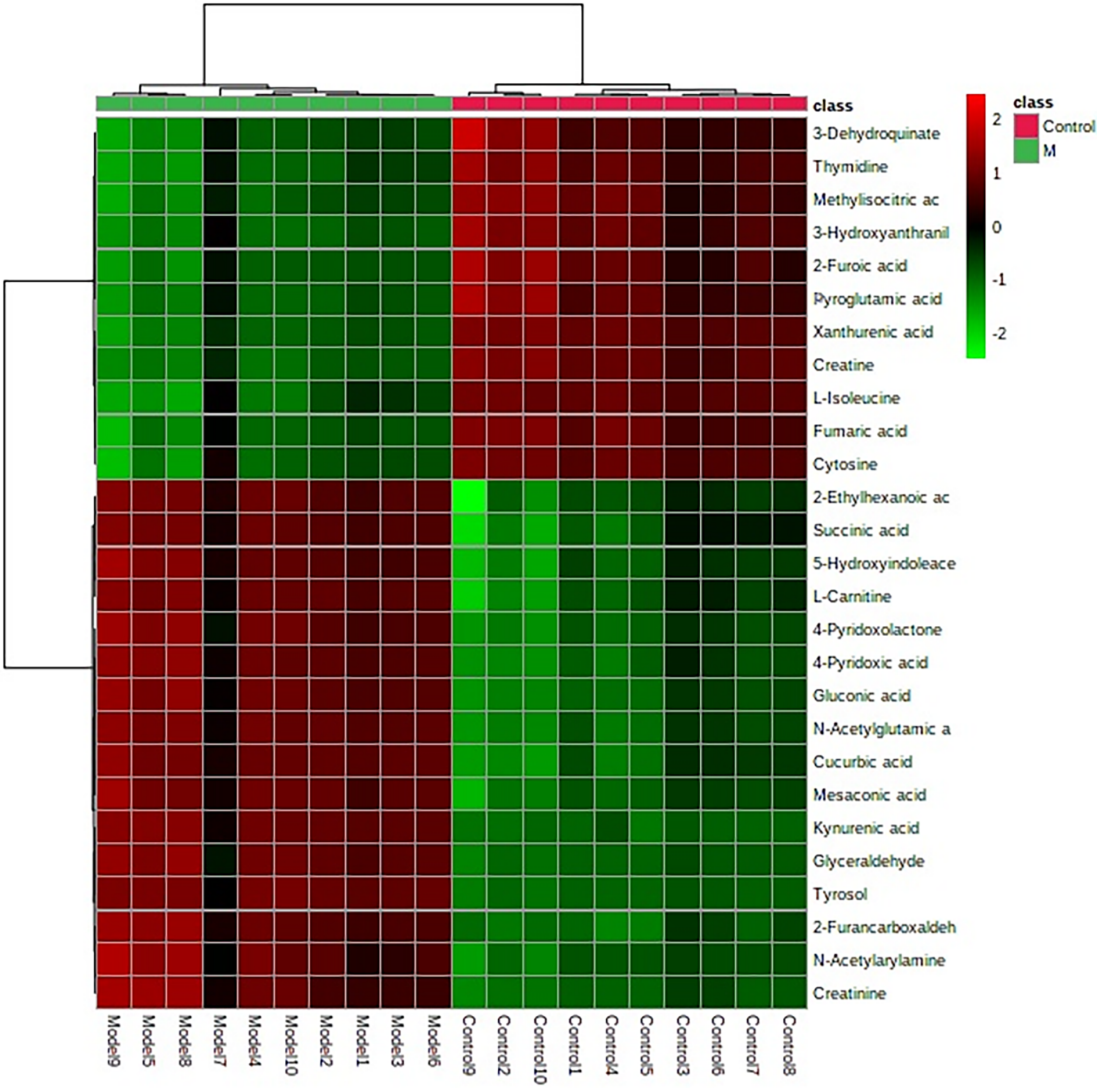
Heatmap clustering for potential biomarkers in the control, and model groups. Individual samples are in columns. Samples are identified by the colored bars on the top. Clustering of the potential biomarkers was performed with the Ward algorithm based on Euclidean distances.

The “.usp” files were processed using MassLynx 4.1 to obtain the score plot under unsupervised mode (**Figures 1**, **2**), and the points in the control group were obviously separated from the model group. Then VIP coefficient of variation was given (**Figure 3**) for screening conditions for the potential biomarkers, which evaluated the contribution of the urine metabolites for intestinal tumors. With the database, such as HMDB, Metlin, Chemspider, and KEGG, the ion information of both MS and MS/MS data matched the known fragment information online, and we gained 27 potential biomarkers associated with intestinal tumors (**Table S1**). These biomarkers were in several pathways (**Figure 4**), including purine metabolism, tyrosine metabolism, tryptophan metabolism, valine, leucine and isoleucine degradation, glycine and serine metabolism, pyrimidine metabolism, arginine and proline metabolism, glutamate metabolism, and fatty acid metabolism, etc (**Figure 5** and **Table S2**). The heatmap analysis showed the correlation of biomarkers between normal mice and APC^min/+^ mice (**Figure 6**), and almost all the samples in the two groups were separated. Each cube had an altered area to represent the different relative intensities of biomarkers in each sample.

### Metabolic Profile Analysis of HNK Against APC^min/+^ Transgenic Mice Model

**Figure 7:**
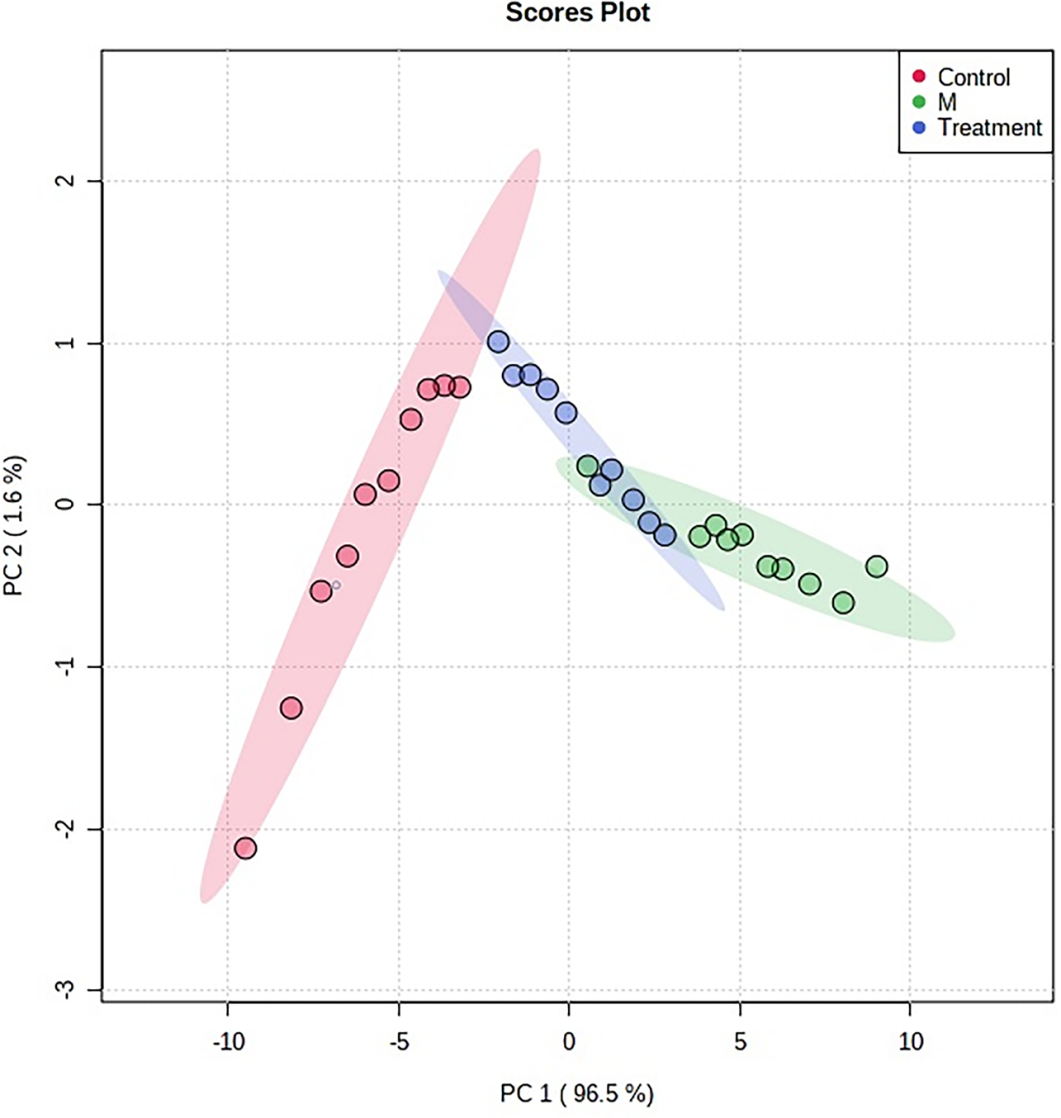
The PCA score plot of control, model group and HNK-treated groups in urine metabolism profile. M, model group. The red dot indicates the control group, the green dot indicates the model group, and the blue dot indicates the treatment group.

**Figure 8:**
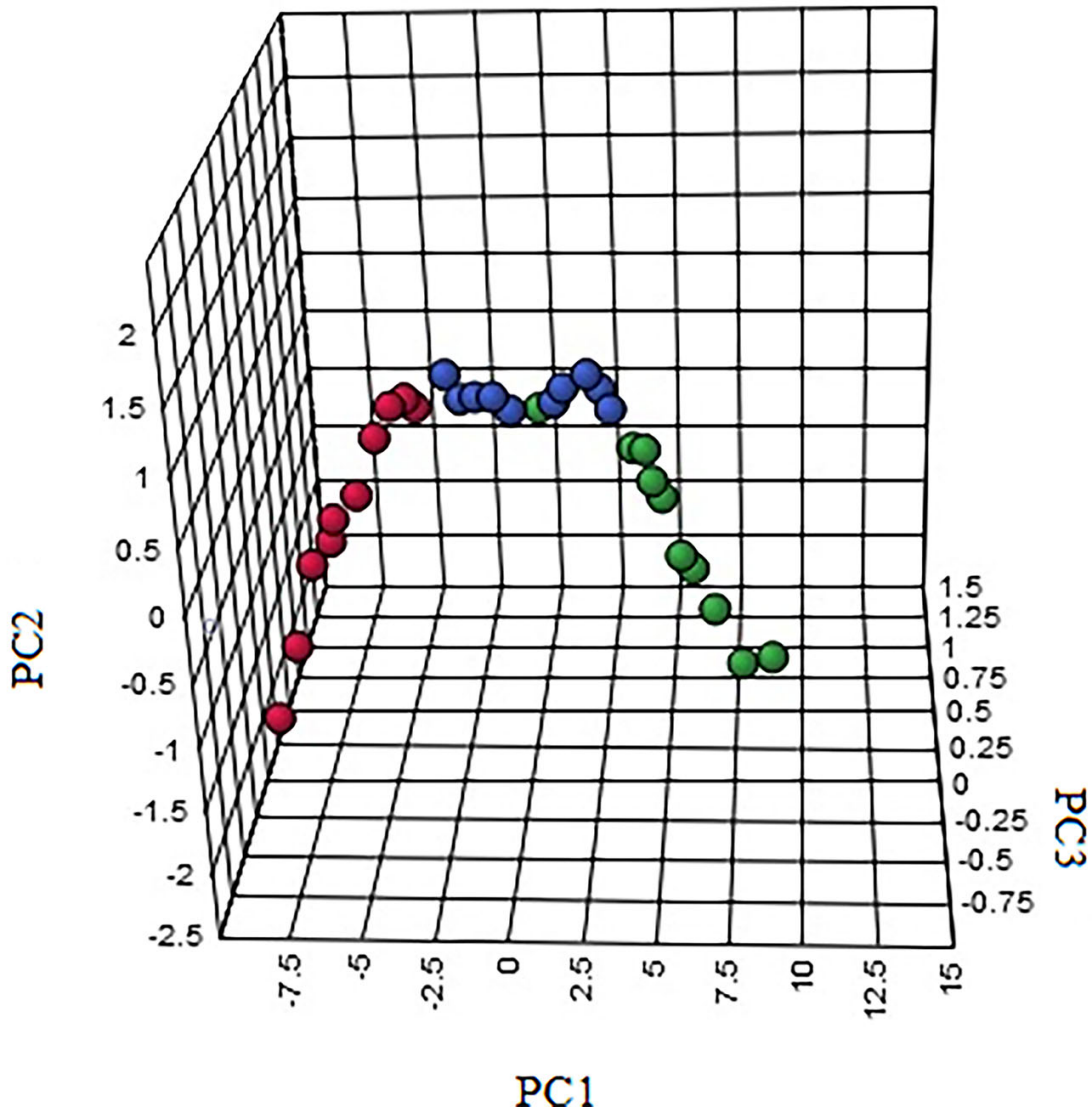
The 3-D PCA score plot of control, model, and HNK-treated groups in urine metabolism profile. M, model group. The red dot indicates the control group, the green dot indicates the model group, and the blue dot indicates the treatment group.

In order to characterize the effects of HNK in our study, unsupervised variable analysis was used to obtain the PCA score of the control, model, and HNK-treated group (**Figures 7**, **8**). Additionally, we could see the control and model groups were divided obviously. The HNK-treated group was located beside the control, model, indicating that there may be some certain difference between the HNK-treated group and the model.

### The Overall Change of Metabolism After the Administration of HNK

**Figure 9:**
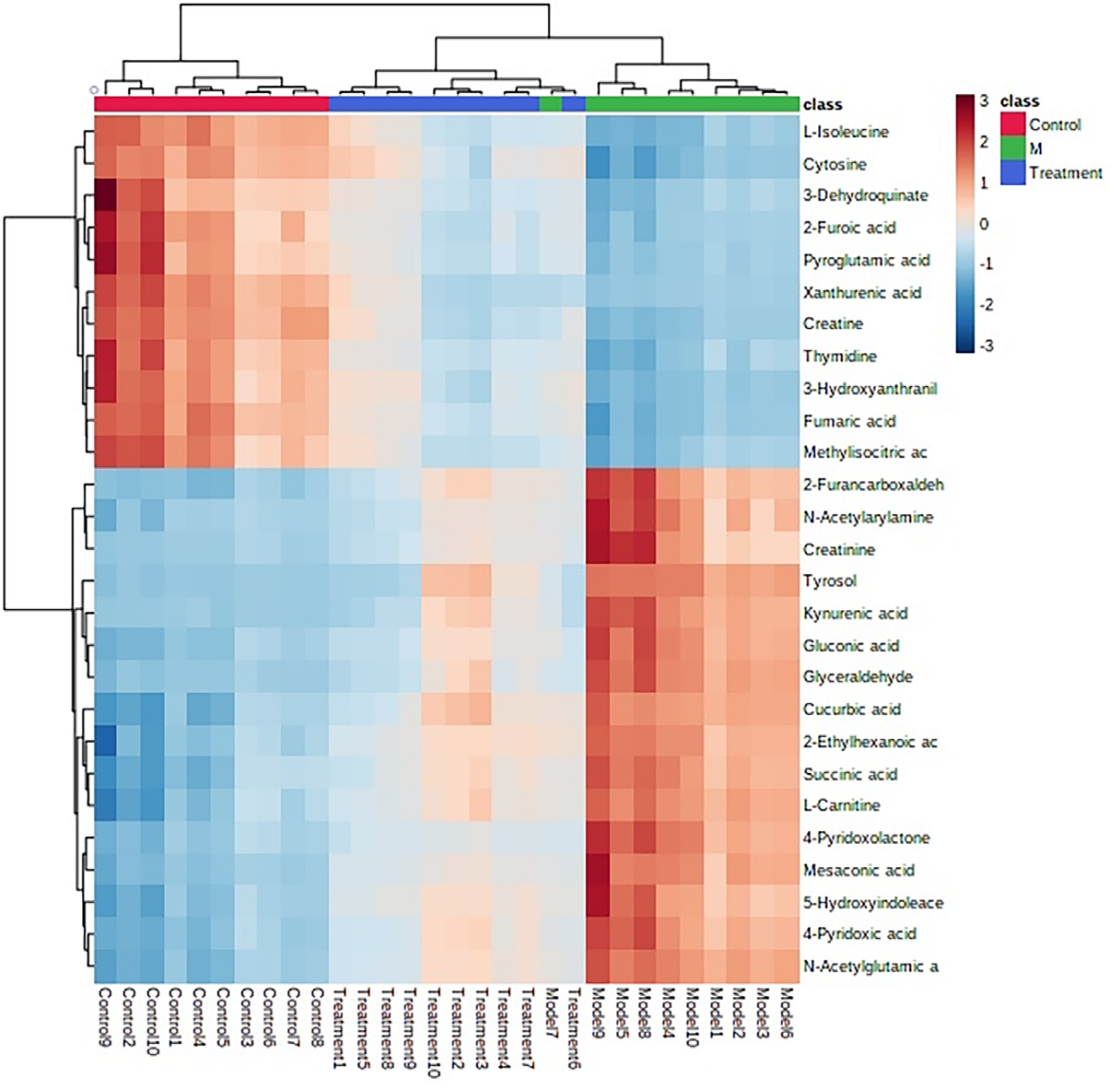
The heatmap analysis for control, model and HNK-treated groups in urine metabolism profile. Heatmap clustering of the potential biomarkers in the different experimental groups. The individual samples are in columns. Samples are identified by the colored bars on the top. Clustering of the potential biomarkers was performed with the Ward algorithm based on Euclidean distances.

**Figure 10:**
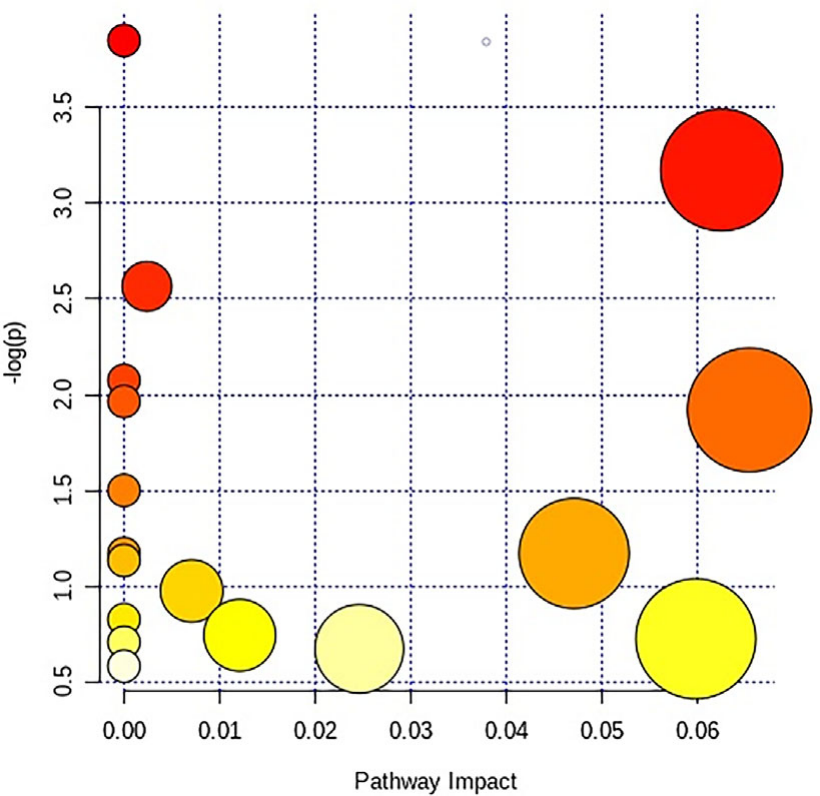
The different canonical pathways regulated by HNK in the current study. The colors refer to the impact score of the metabolic pathways. The identified metabolic pathways are represented as circles, whose color and size are based, respectively, on the *p*-values, calculated with the hypergeometric test (from white to deep red) and on the pathway impact values, from the smallest to the largest. Summary pathway analysis is reported in **Supplementary Table S3**.

After receiving oral administration of HNK for 10 weeks, the biomarkers were significantly regulated (**Figure 9**), which suggested that citrate cycle (TCA cycle), pyrimidine metabolism, pentose phosphate pathway, tyrosine metabolism, arginine and proline metabolism, glutathione metabolism were highly related to HNK-treated effects (**Figure 10** and **Table S3**) by enrichment analysis.

## Discussion

APC^min/+^ mice are the classic model of spontaneous intestinal tumors (21). When the adenoma is formed, the activity of the intestinal lumen is blocked, and the turbid gas is difficult to discharge, thus affecting the overall health. It has been reported that energy metabolism, polyamine metabolism, and composition of intestinal flora are associated with precancerous lesions (22, 23). In this study, we used UPLC-Q-TOF-MS technology to obtain the global metabolomic profiling and utilized the multivariate statistical analysis to receive the potential biomarkers. The profiling of urine metabolism was different between wild-type mice and APC^min/+^ mice, which proved that some metabolisms were disturbed under the condition of disease, and the tryptophan metabolism was one of the key pathways (**Table S3**) from the summary pathway analysis by the MetaboAnalysis 4.0. Through the pathway analysis, HNK mainly managed the tryptophan metabolism, citrate cycle (TCA cycle), and pentose phosphate pathway (**Table S3**), which implied that HNK had a positive correlation with immune function and energy metabolism (24, 25).

Butyrate, a short-chain fatty acid, originates from the fermentation of fiber in the bowel and supplies energy substrates for colonocytes, and the energy of butyrate metabolism depends on colonocytes (26–28). Butyrate decreases its own oxidation in colorectal cancer cells through inhibition of histone deacetylases (29, 30). Patients with CRC have decreased butyrate levels *in vivo*, which is caused by pro-inflammatory cytokines, such as IL-1β (31). Some studies have shown that regulating butyrate metabolism is positive for the treatment of colorectal cancer (32). Another type of metabolism is ketone metabolism, which has been reported to have two main ketone bodies, acetoacetate and 3-hydroxybutyrate, which function in energy transport from the liver to other tissues outside, especially when there is not enough glucose supply (33). However, there is no direct evidence to verify the relationship between ketone metabolism and CRC.

The tryptophan metabolism, a key regulator, connects the immune system of the enteral cavity and intestinal wall barrier (34). Tryptophan and its endogenous metabolites, such as kynurenine, kynurenic acid, and 5-hydroxytryptamine, and intestinal microflora metabolites, such as indoles, affect intestinal immune balance and take part in host immunity (35), and they are also the ligands of the aromatic hydrocarbon receptors (ARH) (36) to mediate the intestinal immunomodulation, intestinal barrier function as well as in intestinal homeostasis (37, 38). In addition, the variation of tryptophan metabolism is closely related to various intestinal diseases, such as bowel syndrome, inflammatory bowel disease (IBD), and colorectal cancer (CRC) (39, 40). Recently, it has also been noted that tryptophan is closely related to the gut microbe that is associated with IBD, Crohn’s disease, and CRC (41–43). In the current study, some biomarkers were disordered in the model mice, such as 4-(2-aminophenyl)-2,4-dioxobutanoic acid, kynurenic acid, 5-hydroxyindoleacetic acid, and xanthurenic acid. It meant that the results of this study were consistent with previous reports because the development of intestinal tumors was accompanied by inflammatory reactions (44). For example, the NMDA receptor, a novel angiogenic tumor endothelial marker in CRC (45), was inhibited by kynurenic acid, which was upregulated by HNK to the normal level. Besides, the release of glutamate is regulated by the NMDA receptor (46), which is involved in alanine, aspartate, and glutamate metabolism. Both ERK (extracellular-signal-regulated protein kinase) and EGTR have a synergistic effect on the occurrence and development of tumors; however, ERK was inhibited in the IPA after administration of HNK, which suggested that the tumorigenesis was postponed (47). Additionally, IPA predicted that pro-inflammation (IL-1β) was inhibited after administration.

As known, serotonin is synthesized in the periphery under the action of tryptophan hydroxylase I (Tph I) in vertebrates, which is mainly expressed in non-neuronal tissues. For instance, intestinal enterochromaffin cells synthesize nearly 90% of peripheral 5-HT (4). In addition, a very small amount of serotonin is also synthesized in the bone tissue (48). Under the action of type A monoamine oxidase and aldehyde dehydrogenase, serotonin is mainly converted to 5-hydroxyindoleacetic acid (5HIAA). It is known that levels of serotonin are also influenced by the tryptophan-degrading enzyme, indoleamine 2,3-dioxygenase, and tetrahydrobiopterin, the cofactor of tryptophan hydroxylase (49). It is reported that 5-HIAA is an important biomarker of several cancers, such as gastric cancer, neuroendocrine tumors (NETs), gastroenteropancreatic neuroendocrine (GEP NET) tumors, *etc* (50–52). In the current study, HNK treatment could regulate the tryptophan metabolism and suggest that the action mechanism of HNK was associated with the repairing of tryptophan metabolites.

## Conclusion

In this study, we provided evidence on the effectiveness of HNK in intestinal tumors in a mouse model (ApcMin/+) using UPLC-Q-TOF/MS technology and multiple statistical analyses, which highlighted significant and potential biomarkers and pharmaceutical pathways. We showed that a total of 27 metabolites as the most contributory biomarkers of intestinal tumors and HNK could affect the regulation of the citric acid cycle, pentose phosphate, pyrimidine metabolic pathway, arginine and proline metabolism, tyrosine metabolism, and glutathione metabolism. It is suggested that the curative effect of HNK is achieved by influencing related metabolic pathways and regulating metabolic functions and affecting multi-pathway systems. The results showed that urine metabolomics can be used as a comprehensive method for evaluating the pharmacological action of natural products.

## Data Availability Statement

The original contributions presented in the study are included in the article/**Supplementary Material**. Further inquiries can be directed to the corresponding author.

## Ethics Statement 

The animal study was reviewed and approved by Animal Care and Ethics Committee of Guang’anmen Hospital, China Academy of Chinese Medical Sciences.

## Author Contributions

XChen contributed to study design, transgenic mice experiments, metabolic pathways and data analysis, and manuscript writing. H-gZ developed the research strategy and conceptualized the manuscript. B-lS and R-zQ reviewed and edited the manuscript. XChang contributed to references preparation and collection. All authors contributed to the article and approved the submitted version.

## Funding

This study was supported by Inherits and Promotion Event of Best and Oldest Nationwide Traditional Chinese Medicine Doctor, entrusted by the National Administration of Traditional Chinese Medicine (GZY-GCS-2018-071), National Natural Science Foundation of China (No. 81673961) and the Beijing Natural Science Foundation (No. 7172186). The funding bodies had no role in study design, subject enrollment, or data analysis.

## Conflict of Interest

The authors declare that the research was conducted in the absence of any commercial or financial relationships that could be construed as a potential conflict of interest.

## Publisher’s Note

All claims expressed in this article are solely those of the authors and do not necessarily represent those of their affiliated organizations, or those of the publisher, the editors and the reviewers. Any product that may be evaluated in this article, or claim that may be made by its manufacturer, is not guaranteed or endorsed by the publisher.
