# Supplementary material for: Ultra-Performance Liquid Chromatography/Mass Spectrometry-Based Metabolomics for Discovering Potential Biomarkers and Metabolic Pathways of Colorectal Cancer in Mouse Model (ApcMin/+) and Revealing the Effect of Honokiol
Source: Front Oncol. 2021 Sep 13;11:671014. doi: 10.3389/fonc.2021.671014 (PMC8473824; doi:10.3389/fonc.2021.671014)
Supplement: Supplementary file 1 [file DataSheet_1.docx]

Table S1 Potential biomarkers of urine sample by ultra-performance liquid chromatography/mass spectrometry metabolomics.

| No | Compound | Compound ID | Mode | Formula | Description | m/z | Retention time (min) |
| --- | --- | --- | --- | --- | --- | --- | --- |
| 1 | 1.28_184.0614m/z | HMDB00017 | M+H | C8H9NO4 | 4-Pyridoxic acid | 184.0614 | 1.28 |
| 2 | 1.96_132.1019m/z | HMDB00172 | M+H | C6H13NO2 | L-Isoleucine | 132.1019 | 1.96 |
| 3 | 5.10_190.0504m/z | HMDB00715 | M+H | C10H7NO3 | Kynurenic acid | 190.0504 | 5.10 |
| 4 | 6.46_111.0438m/z | HMDB33002 | M+H | C6H6O2 | 5-Methyl-2-  furancarboxaldehyde | 111.0438 | 6.46 |
| 5 | 1.87_243.0967m/z | HMDB00273 | M+H | C10H14N2O5 | Thymidine | 243.0967 | 1.87 |
| 6 | 4.01_206.0451m/z | HMDB00881 | M+H | C10H7NO4 | Xanthurenic acid | 206.0451 | 4.01 |
| 7 | 2.38_258.1100m/z | HMDB00086 | M+H | C8H20NO6P | Glycerophosphocholine | 258.11 | 2.38 |
| 8 | 7.41_213.1486m/z | HMDB29388 | M+H | C12H20O3 | Cucurbic acid | 213.1486 | 7.41 |
| 9 | 2.78_166.0504m/z | HMDB03454 | M+H | C8H7NO3 | 4-Pyridoxolactone | 166.0504 | 2.78 |
| 10 | 2.30_132.0774m/z | HMDB00064 | M+H | C4H9N3O2 | Creatine | 132.0774 | 2.30 |
| 11 | 4.29_114.0654m/z | HMDB00562 | M+H | C4H7N3O | Creatinine | 114.0654 | 4.29 |
| 12 | 4.35_162.1134m/z | HMDB00062 | M+H | C7H15NO3 | L-Carnitine | 162.1134 | 4.35 |
| 13 | 1.93_189.0398m/z | HMDB12710 | M-H | C7H10O6 | 3-Dehydroquinate | 189.0398 | 1.93 |
| 14 | 4.76_190.0499m/z | HMDB00763 | M-H | C10H9NO3 | 5-Hydroxyindoleacetic acid | 190.0499 | 4.76 |
| 15 | 8.68_195.0489m/z | HMDB00625 | M-H | C6H12O7 | Gluconic acid | 195.0489 | 8.68 |
| 16 | 6.75_143.1066m/z | HMDB31230 | M-H | C8H16O2 | 2-Ethylhexanoic acid | 143.1066 | 6.75 |
| 17 | 7.46_115.0027m/z | HMDB00134 | M-H | C4H4O4 | Fumaric acid | 115.0027 | 7.46 |
| 18 | 7.80_111.0081m/z | HMDB00617 | M-H | C5H4O3 | 2-Furoic acid | 111.0081 | 7.80 |
| 19 | 8.68_134.0603m/z | HMDB01250 | M-H | C8H9NO | N-Acetylarylamine | 134.0603 | 8.68 |
| 20 | 1.32_129.0189m/z | HMDB00749 | M-H | C5H6O4 | Mesaconic acid | 129.0189 | 1.32 |
| 21 | 1.85_205.0347m/z | HMDB06471 | M-H | C7H10O7 | Methylisocitric acid | 205.0347 | 1.85 |
| 22 | 2.32_188.0556m/z | HMDB01138 | M-H | C7H11NO5 | N-Acetylglutamic acid | 188.0556 | 2.32 |
| 23 | 3.14_117.0187m/z | HMDB00254 | M-H | C4H6O4 | Succinic acid | 117.0187 | 3.14 |
| 24 | 3.92_110.0351m/z | HMDB00630 | M-H | C4H5N3O | Cytosine | 110.0351 | 3.92 |
| 25 | 7.25_152.0349m/z | HMDB01476 | M-H | C7H7NO3 | 3-Hydroxyanthranilic acid | 152.0349 | 7.25 |
| 26 | 7.29_128.0359m/z | HMDB00267 | M-H | C5H7NO3 | Pyroglutamic acid | 128.0359 | 7.29 |
| 27 | 7.83_137.0600m/z | HMDB04284 | M-H | C8H10O2 | Tyrosol | 137.06 | 7.83 |

Table S2 Overview of metabolite sets by the enrichment analysis

| **Name** | **total** | **hits** | **Raw p** |
| --- | --- | --- | --- |
| Purine Metabolism | 74 | 1 | 0.872 |
| Tyrosine Metabolism | 72 | 1 | 0.864 |
| Tryptophan Metabolism | 60 | 4 | 0.0679 |
| Valine, Leucine and Isoleucine Degradation | 60 | 2 | 0.477 |
| Glycine and Serine Metabolism | 59 | 1 | 0.803 |
| Pyrimidine Metabolism | 59 | 1 | 0.803 |
| Warburg Effect | 58 | 2 | 0.459 |
| Arginine and Proline Metabolism | 53 | 3 | 0.16 |
| Glutamate Metabolism | 49 | 1 | 0.739 |
| Fatty acid Metabolism | 43 | 1 | 0.691 |
| Aspartate Metabolism | 35 | 1 | 0.614 |
| Citric Acid Cycle | 32 | 2 | 0.205 |
| Fructose and Mannose Degradation | 32 | 1 | 0.58 |
| Urea Cycle | 29 | 1 | 0.544 |
| Phenylalanine and Tyrosine Metabolism | 28 | 1 | 0.532 |
| Mitochondrial Beta-Oxidation of Long Chain Saturated Fatty Acids | 28 | 1 | 0.532 |
| Mitochondrial Beta-Oxidation of Short Chain Saturated Fatty Acids | 27 | 1 | 0.519 |
| Oxidation of Branched Chain Fatty Acids | 26 | 2 | 0.148 |
| Phytanic Acid Peroxisomal Oxidation | 26 | 1 | 0.505 |
| Glycerolipid Metabolism | 25 | 1 | 0.491 |
| Carnitine Synthesis | 22 | 2 | 0.112 |
| Glutathione Metabolism | 21 | 1 | 0.433 |
| Vitamin B6 Metabolism | 20 | 1 | 0.417 |
| Mitochondrial Electron Transport Chain | 19 | 2 | 0.0869 |
| Butyrate Metabolism | 19 | 1 | 0.401 |
| Beta Oxidation of Very Long Chain Fatty Acids | 17 | 1 | 0.367 |
| Ketone Body Metabolism | 13 | 1 | 0.295 |

Note: Total is the total number of compounds in the pathway; the Hits is the actually matched number from the user uploaded data; the Raw pis the original p value calculated from the enrichment analysis.

Table S3 Summary pathway analysis by the MetaboAnalysis 4.0

|  | Total | Hits | Raw p | Impact |
| --- | --- | --- | --- | --- |
| Tryptophan metabolism | 41 | 2 | 0.15 | 0.07 |
| Citrate cycle (TCA cycle) | 20 | 2 | 0.04 | 0.06 |
| Pyrimidine metabolism | 39 | 1 | 0.48 | 0.06 |
| Pentose phosphate pathway | 22 | 1 | 0.31 | 0.05 |
| Tyrosine metabolism | 42 | 1 | 0.51 | 0.02 |
| Arginine and proline metabolism | 38 | 1 | 0.47 | 0.01 |
| Glutathione metabolism | 28 | 1 | 0.38 | 0.01 |
| Alanine, aspartate and glutamate metabolism | 28 | 2 | 0.08 | 0.00 |
| Arginine biosynthesis | 14 | 2 | 0.02 | 0.00 |
| Valine, leucine and isoleucine biosynthesis | 8 | 1 | 0.13 | 0.00 |
| Vitamin B6 metabolism | 9 | 1 | 0.14 | 0.00 |
| Butanoate metabolism | 15 | 1 | 0.22 | 0.00 |
| Pyruvate metabolism | 22 | 1 | 0.31 | 0.00 |
| Propanoate metabolism | 23 | 1 | 0.32 | 0.00 |
| Glycine, serine and threonine metabolism | 34 | 1 | 0.44 | 0.00 |
| Valine, leucine and isoleucine degradation | 40 | 1 | 0.49 | 0.00 |
| Aminoacyl-tRNA biosynthesis | 48 | 1 | 0.56 | 0.00 |

Note: Total is the total number of compounds in the pathway; the Hits is the actually matched number from the user uploaded data; the Raw pis the original p value calculated from the enrichment analysis; the Impact is the pathway impact value calculated from pathway topology analysis.


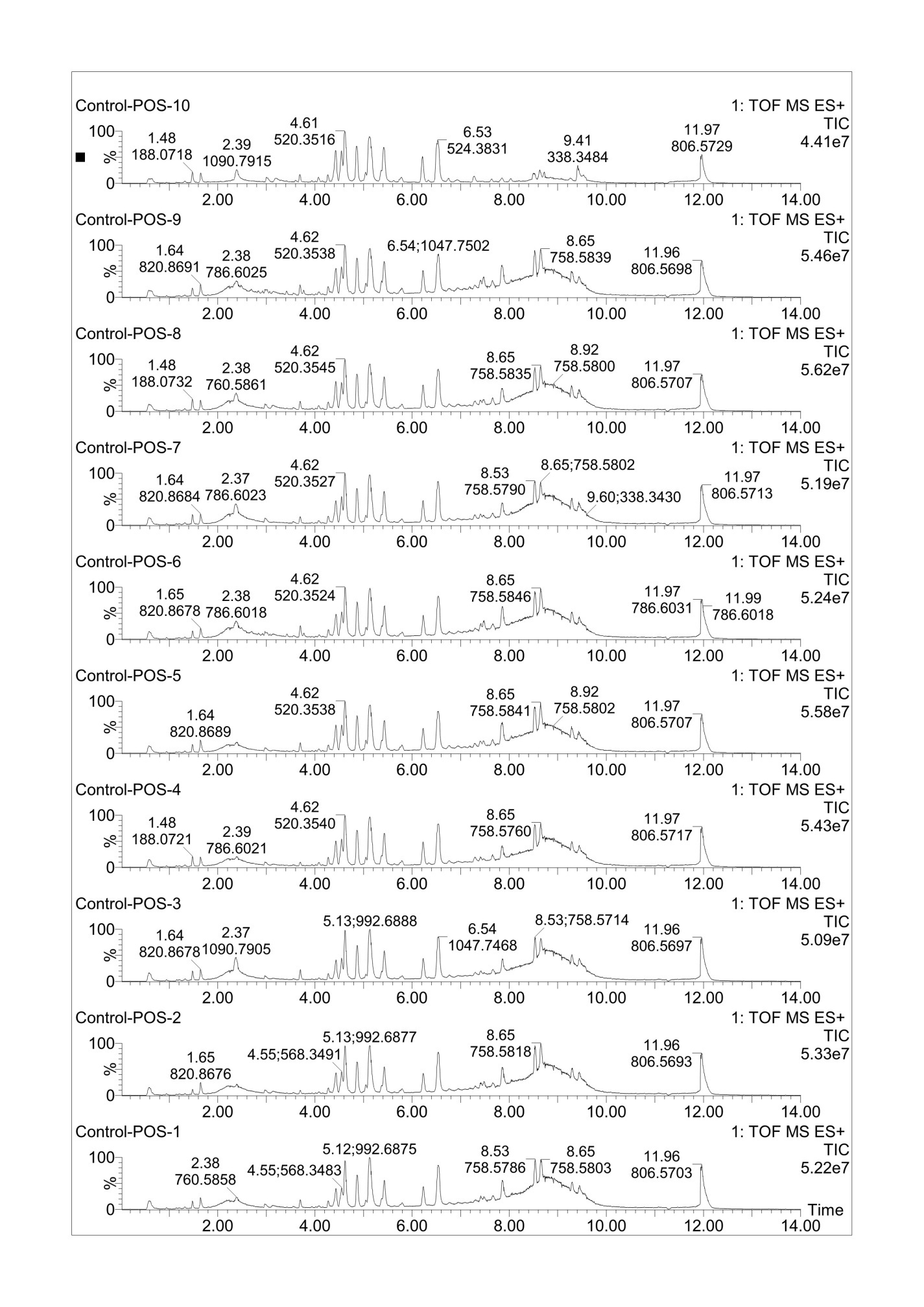


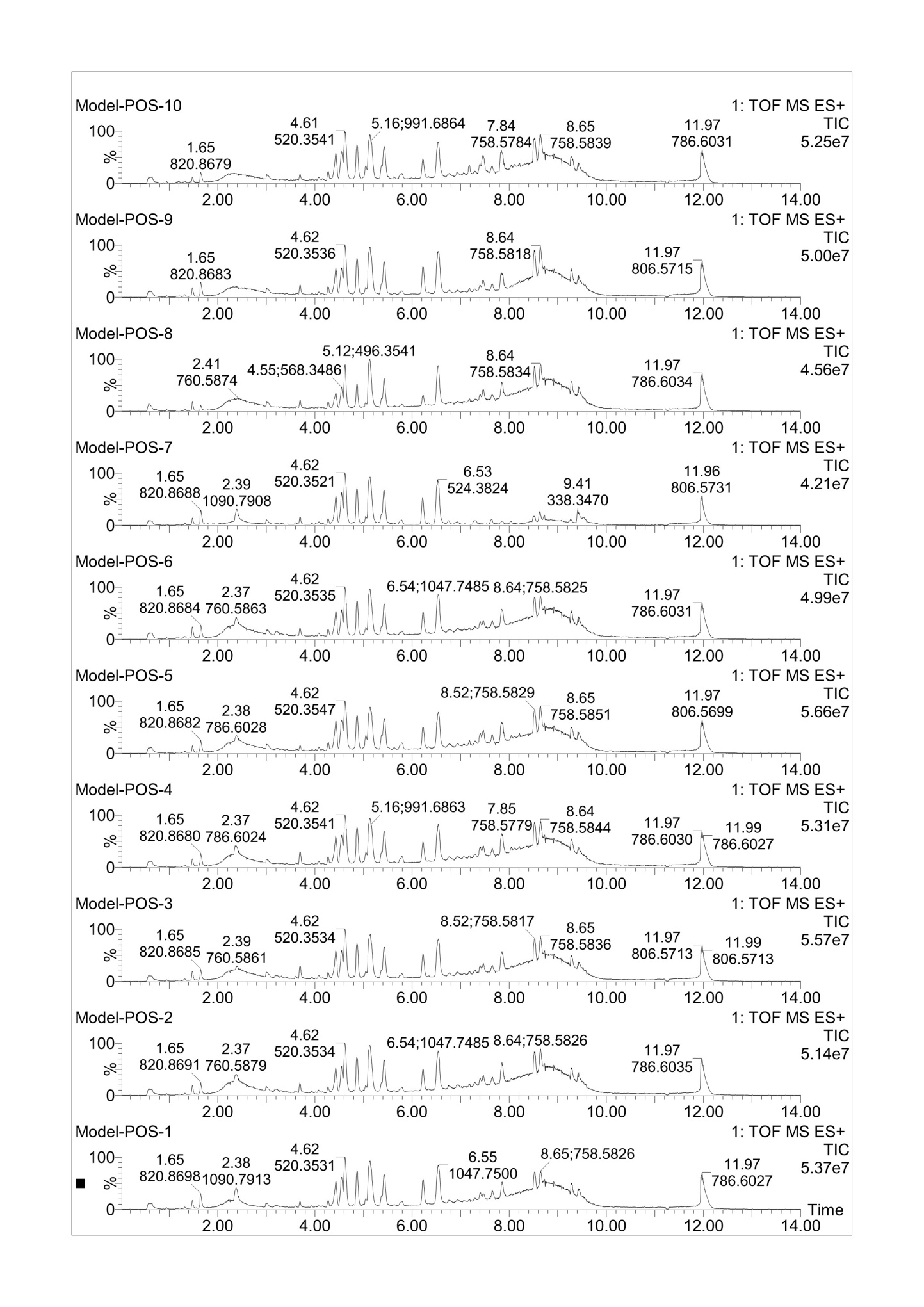


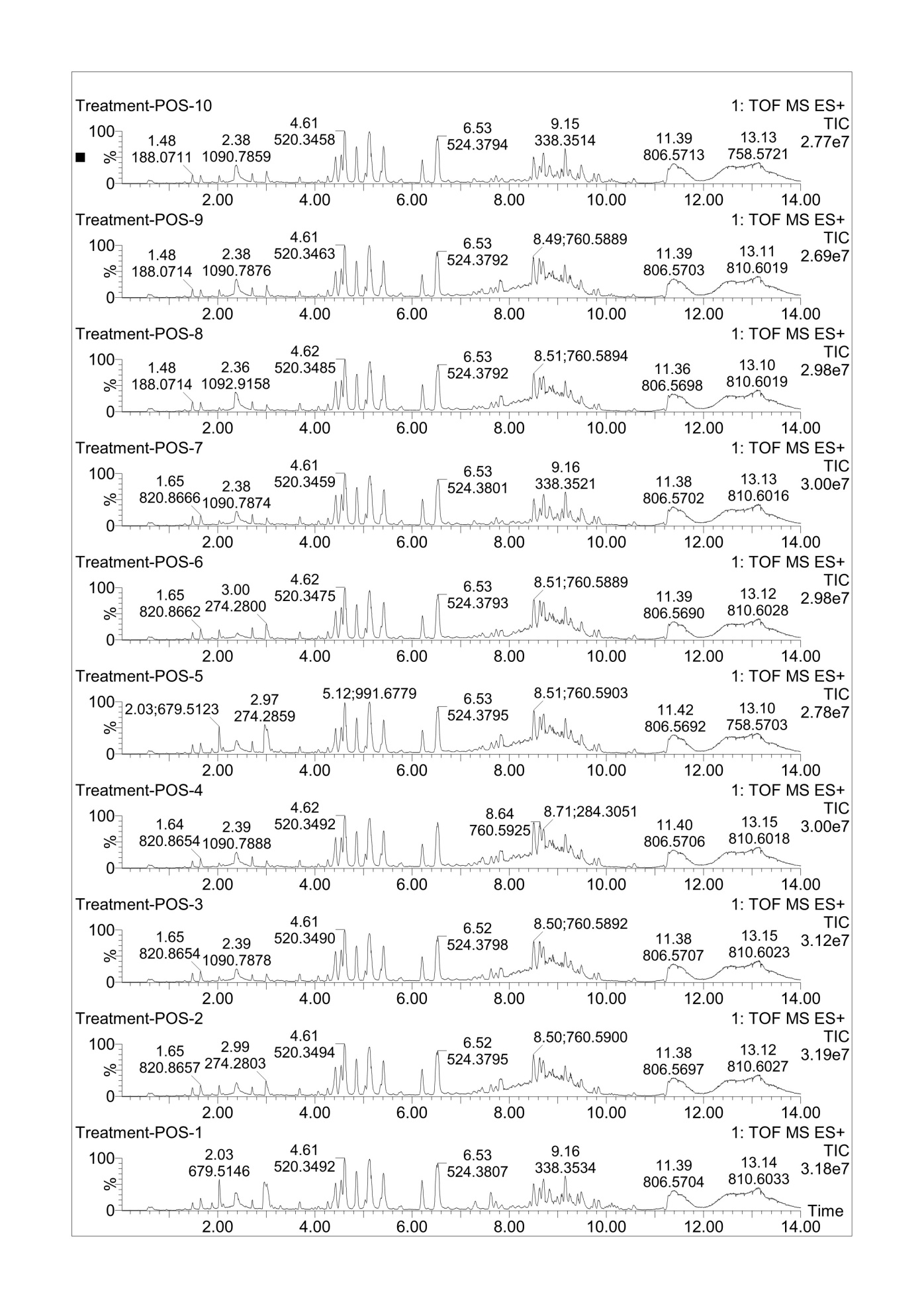


Figure S1. The chromatograms of the ultra-high-performance liquid chromatography in the positive ion mode.


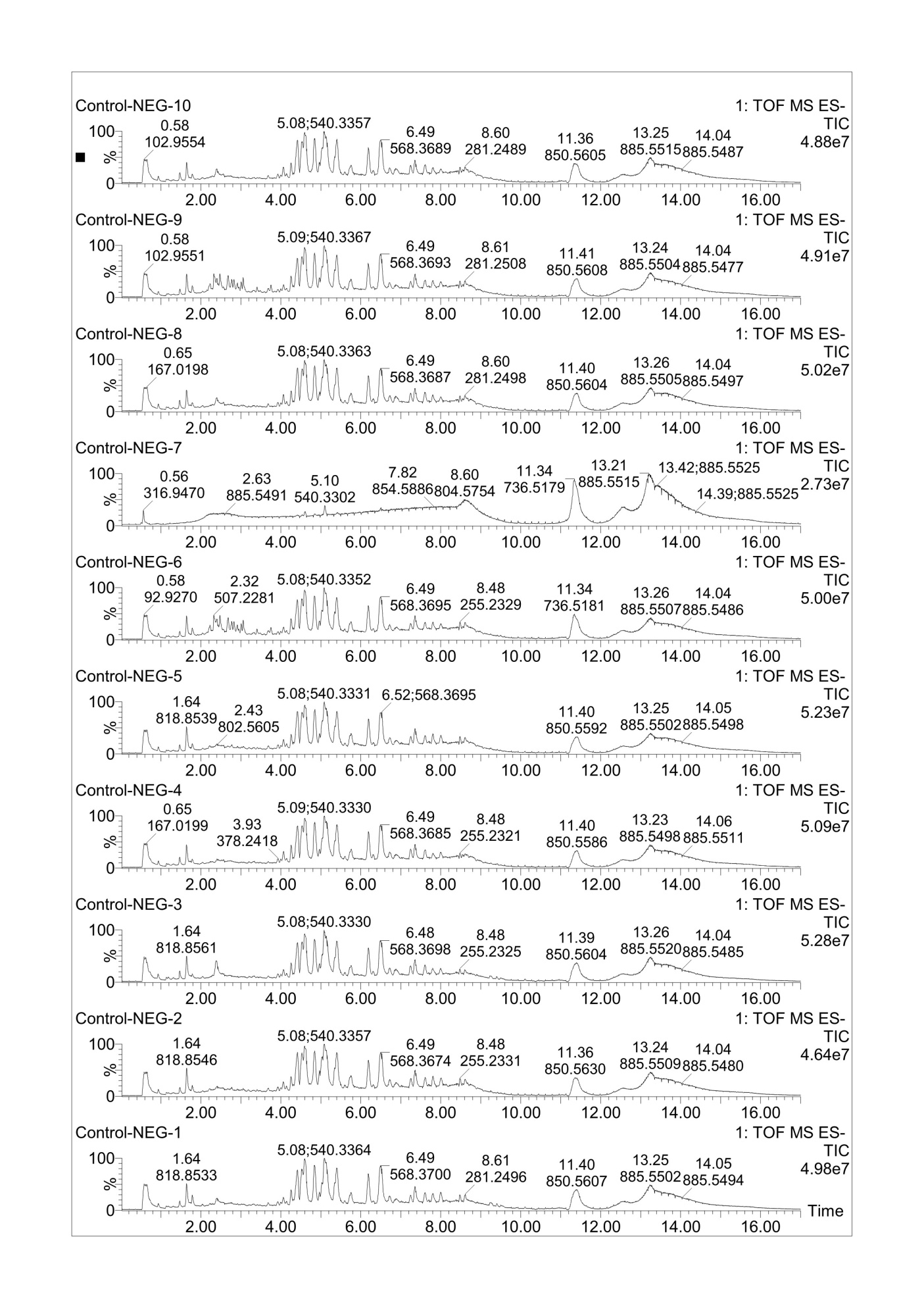


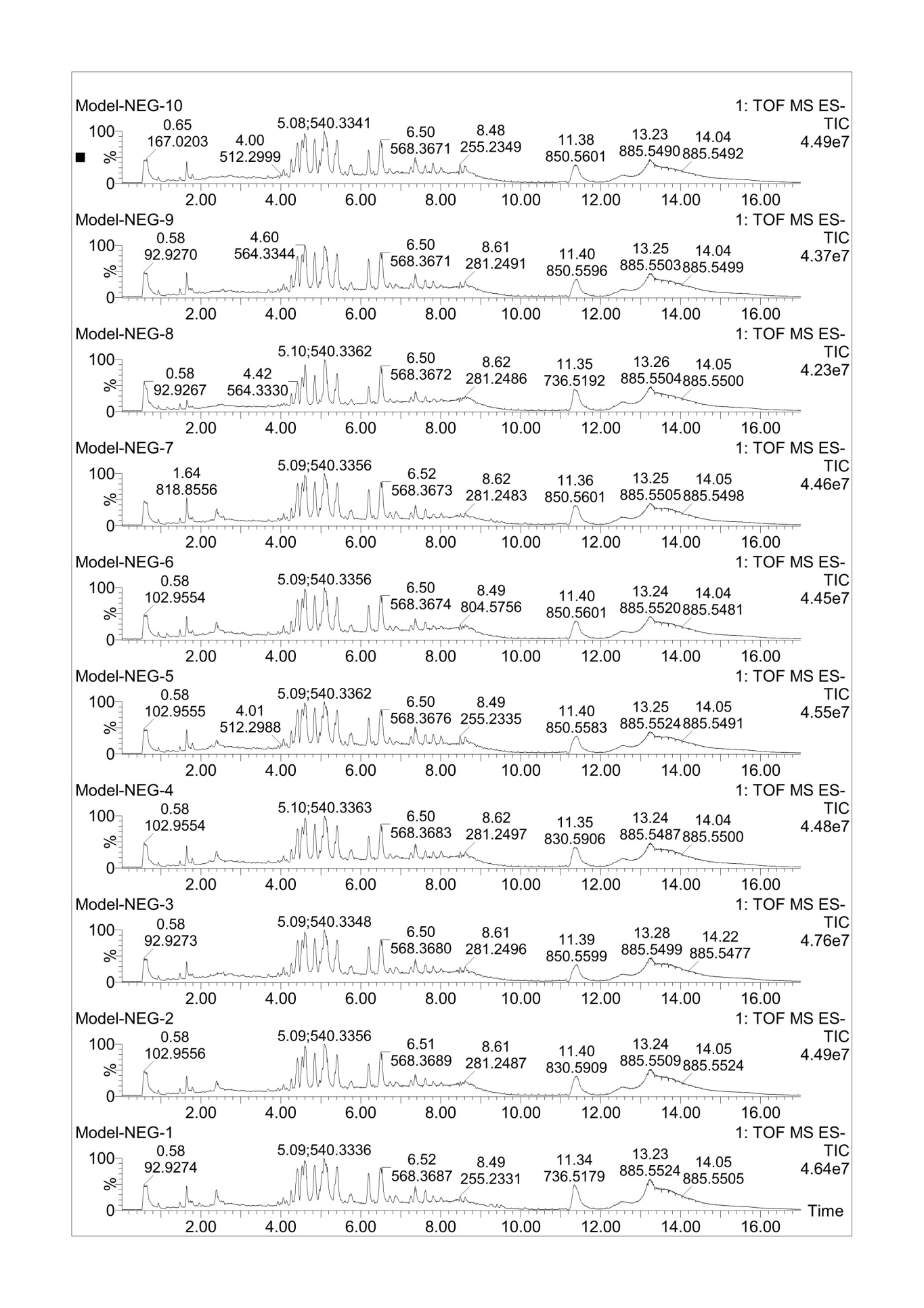


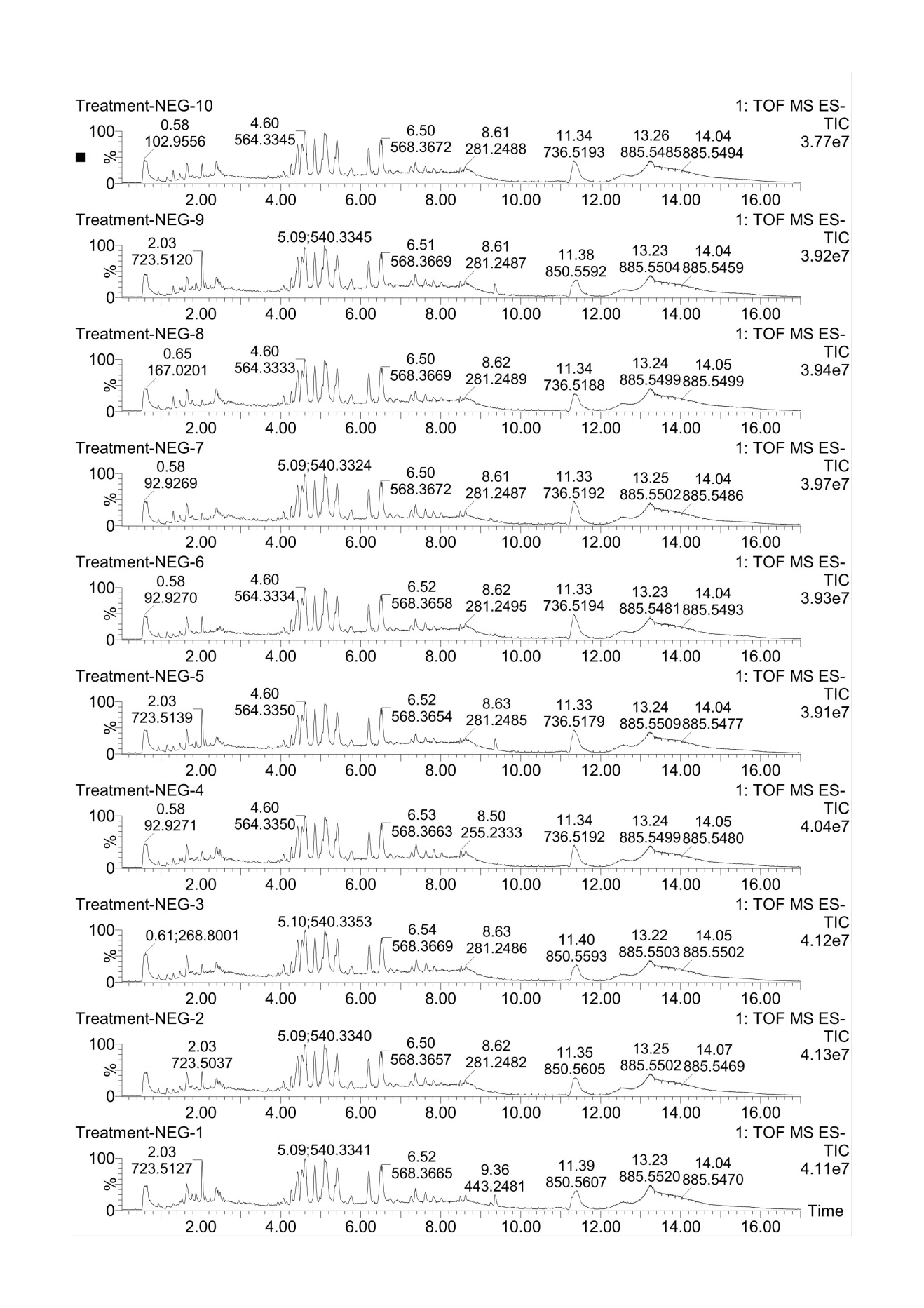


Figure S2. The chromatograms of the ultra-high-performance liquid chromatography in the negative ion mode.
